# Computational Simulator Models and Invasive Hemodynamic Monitoring as Tools for Precision Medicine in Pulmonary Arterial Hypertension

**DOI:** 10.3390/jcm11010082

**Published:** 2021-12-24

**Authors:** Giovanna Manzi, Cristiano Miotti, Marco Valerio Mariani, Silvia Papa, Federico Luongo, Gianmarco Scoccia, Beatrice De Lazzari, Claudio De Lazzari, Raymond L. Benza, Francesco Fedele, Carmine Dario Vizza, Roberto Badagliacca

**Affiliations:** 1 Department of Clinical, Anesthesiological and Cardiovascular Sciences, Sapienza University of Rome, Viale del Policlinico, 155, 00161 Rome, Italy; giovannamanzi91@gmail.com (G.M.); miotticristiano@gmail.com (C.M.); marcoval.mariani@gmail.com (M.V.M.); silviapapa83@gmail.com (S.P.); federico.luongo91@gmail.com (F.L.); gianmarcoscoccia@gmail.com (G.S.); francesco.fedele@uniroma1.it (F.F.); dario.vizza@uniroma1.it (C.D.V.); 2 Department of Engineering, Roma Tre University, 00146 Rome, Italy; beatrice.delazzari@gmail.com; 3 National Research Council, Institute of Clinical Physiology (IFC-CNR), 00185 Rome, Italy; claudio.delazzari@ifc.cnr.it; 4 Division of Cardiovascular Diseases, Ohio State University, Columbus, OH 43210, USA; Raymond.Benza@ahn.org

**Keywords:** implantable hemodynamic monitors, computational models, pulmonary arterial hypertension, precision medicine, big data

## Abstract

Precision medicine, providing the right therapeutic strategy for the right patient, could revolutionize management and prognosis of patients affected by cardiovascular diseases. Big data and artificial intelligence are pivotal for the realization of this ambitious design. In the setting of pulmonary arterial hypertension (PAH), the use of computational models and data derived from ambulatory implantable hemodynamic monitors could provide useful information for tailored treatment, as requested by precision medicine.

## 1. Introduction

Advances in panomics, including genomics, proteomics, transcriptomics and metabolomics, are paving the way for the shift from population-based clinical decision making to a true personalization of care based on individual genetic and environmental factors. Despite the success achieved with medical reductionism, the idea that all patients with the same disease should be treated similarly is too limited to be accepted today [1]. Treating the patient, not the disease is the goal for precision medicine.

In spite of advances achieved with drugs targeting the endothelin, nitric oxide and prostacyclin pathways involved in the disease, PAH is still characterized by high morbidity and mortality rates [2,3]. Thus, the current challenge is the development of personalized medicine strategies, providing the right treatment to the right patient [4].

**Figure 1:**
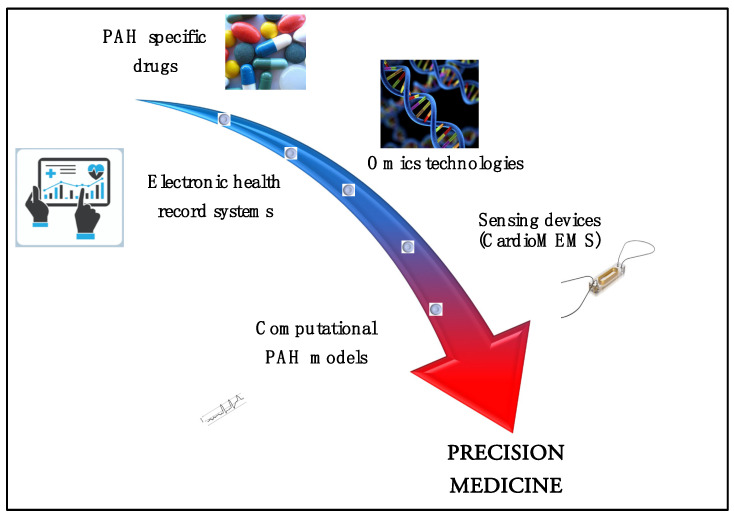
Hypothetical pathway for precision medicine in PAH. Treating the patient, not the disease, is the goal for precision medicine. Panomics, phenotyping, big data derived from electronical health systems and sensing devices, and artificial intelligence with the use of computational simulators could contribute to developing this ambitious goal in PAH, overcoming the current limitations of reductionism.

Omic technologies, big data and artificial intelligence could contribute to realizing this ambitious goal, allowing a step forward in current clinical practice (Figure 1).

As the right ventricle (RV) is the main determinant of patient’s prognosis [5], computational models simulating the effects of increased afterload on RV function and predicting response to specific drugs could aid physicians in optimizing PAH understanding, prognosis and clinical decision making. Versatility, reliability, simplicity and low execution time are pivotal for the success of these models.

The present paper focuses on new potential opportunities offered by computational models and data derived from ambulatory implantable hemodynamic monitoring (IHM), used to provide useful information for PAH patient management, as expected in the precision medicine era.

## 2. A Look at Omic Technologies in PAH

The current World Symposium on Pulmonary Hypertension (WSPH) classification categorizes patients on the basis of similar clinical and hemodynamic patterns without considering differences in genomics, proteomics, transcriptomics and metabolomics. Indeed, only two of the five categories, PAH and pulmonary veno-occlusive disease/pulmonary capillary haemangiomatosis, are linked to molecular mechanism. Similarly, current PAH treatment strategies primarily target pulmonary vasoconstriction, without taking into account recent advances in omics fields. Omic technologies could overcome these limitations and enhance clinical research in PAH, generating new understanding of the disease and improving both diagnosis and treatment.

The description of all omics technologies goes beyond the aim of this perspective paper; however, we choose briefly mention genomics.

The most common genetic cause of PAH involves the gene encoding the bone morphogenetic protein receptor type II (BMPR2), a receptor for the transforming growth factor (TGF)-ß protein superfamily, particularly expressed on the pulmonary vascular endothelium. The reduction in expression or function of BMPR2 signaling leads to altered cellular responses to TGF-ß.

Since the identification of BMPR2 mutations, the PAH genetic landscape has significantly expanded, with novel causative genes detected by new-generation sequencing methodologies (e.g., SMAD9, CAV1, EIF2AK4, ENG) [6]. Genomics advances could lead to progress in PAH therapy through the development of new drugs targeted to mutational correction.

To achieve a comprehensive phenotyping of PH patients allowing a more efficacious approach of the PH classification in terms of clinical impact, the National Institutes of Health (NIH)/National Heart, Lung and Blood institute (NHLBI) recently launched an ongoing initiative called “Redefining pulmonary Hypertension through Pulmonary Vascular Diseas Phenomics (PVDOMICS)” [7,8]. To date, the study has enrolled approximately 1200 adult participants with PH, classified according to the traditional WSPH groups one to five, or with risk factors for PH or healthy control individuals. All participants underwent a deep clinical, imaging and hemodynamic assessment and blood analysis through samples obtained from the peripheral vein, pulmonary artery and distal-to-pulmonary artery occlusion for a broad collection of selected omics tests. The large dataset deriving from PVDOMICS, including clinical and omic biobanks, will represent a precious and unique resource available for future PH investigations.

## 3. Ambulatory Implantable Hemodynamic Monitors: A New Source of Big Data

Big data, referring to the storage and analysis of large volumes of medical information from individual patient data, provide a unique opportunity to improve health, identify personalized therapeutic options and detect different treatment responses to therapy [9]. The main sources of big data include: (a) the electronic patient-health-record systems (EHR), allowing physicians to store, process and share electronically patients’ medical data for the coordination of multicenter registries and (b) sensing devices, wearable and implantable devices for the continuous monitoring of specific parameters [10]. The huge amount of data derived from EHR and sensing devices has radically changed the management of PAH over the last two decades, as well as other cardiovascular diseases.

## 4. Novel Devices for Monitoring Pulmonary Arterial Hypertension Patients

PAH is an obstructive pulmonary vasculopathy characterized by a progressive increase in pulmonary vascular resistance (PVR) that leads to RV overload, and ultimately, RV failure. The close monitoring of pulmonary arterial pressure and RV function could help clinicians tailor the best therapeutic strategy in order to improve patients’ outcome and reduce hospitalization rates [11,12]. Indeed, PAH patients’ functional status and prognosis mostly depend on the ability of the RV to adapt to the increased afterload [13]. In the earlier stages, the increased RV contractility, with little or no increase in right heart chamber dimensions, allows for preserving cardiac output. In the later stages of the disease, the homeometric adaptation fails and the stroke volume is maintained by progressive increase in RV end-diastolic volume (heterometric adaptation) [14,15]. The consequent tricuspid functional regurgitation leads to additional RV volume overload. Finally, the leftward displacement of the interventricular septum and the left ventricle (LV) underfilling due to reduced RV cardiac output accounts for the heart failure (HF) syndrome [16].

In this scenario, optimal management of PAH patients strongly relies on the assessment of RV function, RV remodeling and hemodynamic status [17,18,19]. Echocardiography is the first-line technique for the evaluation of RV size and function [20,21], but does not provide robust hemodynamic data. Conversely, right heart catherization allows for a comprehensive hemodynamic assessment, but it is an invasive procedure with technical challenges, and it must be performed in a clinic, providing a snapshot of patient’s hemodynamics at a single time point. Ambulatory IHMs, such as CardioMEMS^TM^ (Abbott, Sylmar, CA, USA), providing frequent remote hemodynamic measurements in the home setting and recording the variability of pulmonary pressure during the day, provide additional information for PAH patients’ management, potentially overcoming the above limitations [22].

## 5. The CardioMEMS System

IHMs have recently been approved for the management of patients with HF [23], aiding physicians to adjust HF therapy before the development of congestive symptoms [24]. As recommended by the 2016 ESC Heart Failure Guidelines [25], the wireless CardioMEMS^TM^ system may be considered (class IIb) for the monitoring of pulmonary artery pressure in symptomatic patients with HF with previous HF hospitalization, in order to reduce the risk of recurrent HF hospitalization.

Conversely, the experience of IHMs in the management of patients with PAH is still limited to small series. CardioMEMS system implantation in 26 PAH patients with NYHA class III or IV has recently shown that IHMs may represent a promising tool to early identify decompensation or noncompliance, to reassess response to therapy and to allow rapid drug titration, reducing in-person visits and invasive hemodynamic assessments [22,26]. Importantly, while clinical experience with IHMs in PAH is increasing, providing a huge amount of data, IHMs represent a unique opportunity to improve and validate computational cardiovascular simulators in this setting. However, randomized, larger clinical trials are needed before these devices can be routinely used in clinical practice.

## 6. Computational Cardiovascular Simulators

Advances in technology have led to the development of computational simulators, which are useful in research, clinical and e-learning environmental settings [27].

In the field of cardiovascular diseases, the most used numerical simulators provide a representation of the left and right heart, systemic, pulmonary and coronary circulation, relying on pressure–volume relationships and lumped-parameter models [28,29,30].

Computational simulators are intended as interactive software for reproducing physiological and pathological clinical conditions. Usually these are characterized by a modular approach, consisting of a basic core module and supplementary, highly specialized modules. As an example, we may consider the following supplementary modules:

- Supplementary modules for ventricular assist devices;
- A supplementary module to reproduce the biventricular pacemaker behavior;
- A supplementary module for the total artificial heart;
- A supplementary module for intra-aortic balloon pumping;
- A supplementary module for the thoracic artificial lung.

Combining these modules, clinicians may virtually reproduce patient’s hemodynamic conditions, simulate the effects of different therapeutic interventions in real time and obtain accurate prediction of device performance. Thus, such computational simulators (e.g., CARDIOSIM, CircAdapt Simulator, HemoLab and Harvi) could support physicians in clinical decision making [31,32,33,34].

Computational simulators might also play a pivotal role in unravelling the cardiopulmonary unit response to different therapeutic strategies in the complex PAH pathophysiology. Indeed, computational simulators accurately describing the interaction between the respiratory and the circulatory system might correctly reproduce the hemodynamic and respiratory condition of PAH patients, allowing the right drug for the right patient. Nevertheless, only few systems currently include hemodynamic and ventilatory variables coupling the cardiovascular and respiratory system, allowing a complete simulation of PAH pathophysiology. The optimization and the widespread diffusion of computational PAH simulators in daily clinical practice require strict cooperation between model developers and PAH specialists.

## 7. Conclusions

The exponential growth in technology may add a significant contribution to patients’ management and potentially improve the prognosis of patients affected by cardiovascular diseases such as PAH. Reproducing pathophysiological conditions and simulating the effects of drugs and interventional procedures on the cardiovascular system, computational simulators may become a new approach in clinical practice. The validation of these systems, useful for their strength and clinical applications, remains a major issue. Big data derived from electronic patient-health-record systems and ambulatory IHMs could be used to improve and validate computational PAH simulators, welcoming precision medicine era into the PAH field.

## Data Availability

Not applicable.
